# Supplementary material for: Implementation of mobile-health technology is associated with five-year survival among individuals in rural areas of Indonesia
Source: PLOS Digit Health. 2024 Apr 2;3(4):e0000476. doi: 10.1371/journal.pdig.0000476 (PMC10986960; doi:10.1371/journal.pdig.0000476)
Supplement: S1 Table — (DOCX) [file pdig.0000476.s001.docx]

**S1 Table** Baseline characteristics of study participants

|  |  |  | **Intervention villages** | |  |  |  |  | **Control villages** | |  |
| --- | --- | --- | --- | --- | --- | --- | --- | --- | --- | --- | --- |
|  | **Total**  **(n=11,098)** | **Non death**  **(n=10,823)** | **Cardiovascular death**  **(n=169)** | **Non cardiovascular death**  **(n=106)** |  | **P value*** | **Total**  **(n=10,981)** | **Non death**  **(n=10.619)** | **Cardiovascular death**  **(n=252)** | **Non cardiovascular death**  **(n=110)** | **P value*** |
| Age, mean (sd) | 54.71 (10.57) | 54.50 (10.46) | 62.86 (12.16) | 61.86 (10.60) |  | <0.001 | 55.09 (10.94) | 54.80 (10.73) | 63.77 (13.25) | 63.22 (13.98) | <0.001 |
| Female, n (%) | 6,426 (57.92) | 6,288 (58.11) | 90 (53.25) | 48 (45.28) |  | 0.013 | 6,075 (55.32) | 5,903 (55.59) | 122 (48.41) | 50 (45.45) | 0.009 |
| Senior high school or higher degree, % | 2,990 (26.95) | 2,939 (27.16) | 32 (18.93) | 19 (17.92) |  | 0.006 | 2,286 (20.82) | 2,246 (21.15) | 23 (9.13) | 17 (15.45) | <0.001 |
| Married, % | 9,012 (81.23) | 8,806 (81.39) | 127 (75.15) | 79 (74.53) |  | 0.025 | 8,968 (81.67) | 8,729 (82.20) | 164 (65.08) | 75 (68.18) | <0.001 |
| Unemployed, % | 3,880 (34.97) | 3,751 (34.67) | 78 (46.15) | 51 (48.11) |  | <0.001 | 3,584 (32.64) | 3,410 (32.11) | 119 (47.22) | 55 (50.00) | <0.001 |
| Vigorous physical activity, % | 2,915 (26.26) | 2,863 (26.46) | 30 (17.75) | 22 (20.75) |  | 0.017 | 2,701 (24.60) | 2,635 (24.81) | 44 (17.46) | 22 (20.00) | <0.001 |
| BMI, mean (sd) | 25.35 (6.27) | 25.38 (6.23) | 24.38 (8.69) | 23.68 (5.46) |  | <0.001 | 25.07 (5.93) | 25.12 (5.75) | 23.00 (8.59) | 25.10 (12.06) | <0.001 |
| Obese, % | 1,601 (14.50) | 1,563 (14.51) | 26 (15.66) | 12 (11.65) |  | 0.652 | 1,479 (13.55) | 1,449 (13.72) | 17 (6.88) | 13 (12.15) | 0.007 |
| Diabetes, % | 931 (8.39) | 886 (8.19) | 27 (15.98) | 18 (16.98) |  | <0.001 | 715 (6.51) | 675 (6.36) | 28 (11.11) | 12 (10.91) | 0.002 |

**Note:**  The bivariate analysis war performed using chi-square test for categorical and Kruskal Wallis for continuous variables.
