# Supplementary material for: Implementation of mobile-health technology is associated with five-year survival among individuals in rural areas of Indonesia
Source: PLOS Digit Health. 2024 Apr 2;3(4):e0000476. doi: 10.1371/journal.pdig.0000476 (PMC10986960; doi:10.1371/journal.pdig.0000476)
Supplement: S2 Table — (DOCX) [file pdig.0000476.s002.docx]

**S2 Table** Baseline characteristics of study participants in intervention and control villages

|  | **Intervention villages**  **(n=11,098)** | **Control villages**  **(n=10,981)** | **P value*** |
| --- | --- | --- | --- |
| Age, mean (sd) | 54.71 (10.57) | 55.09 (10.94) | 0.054 |
| Female, n (%) | 6,426 (57.92) | 6,075 (55.32) | <0.001 |
| Senior high school or higher degree, % | 2,990 (26.95) | 2,286 (20.82) | <0.001 |
| Married, % | 9,012 (81.23) | 8,968 (81.67) | 0.397 |
| Unemployed, % | 3,880 (34.97) | 3,584 (32.64) | <0.001 |
| Vigorous physical activity, % | 2,915 (26.26) | 2,701 (24.60) | 0.005 |
| BMI, mean (sd) | 25.35 (6.27) | 25.07 (5.93) | <0.001 |
| Obese, % | 1,601 (14.50) | 1,479 (13.55) | 0.043 |
| Diabetes, % | 931 (8.39) | 715 (6.51) | <0.001 |

**Note:**  The bivariate analysis was performed using the chi-square test for categorical and Kruskal Wallis for continuous variables.
