# Supplementary figures and images for: Implementation of mobile-health technology is associated with five-year survival among individuals in rural areas of Indonesia
Source: PLOS Digit Health. 2024 Apr 2;3(4):e0000476. doi: 10.1371/journal.pdig.0000476 (PMC10986960; doi:10.1371/journal.pdig.0000476)

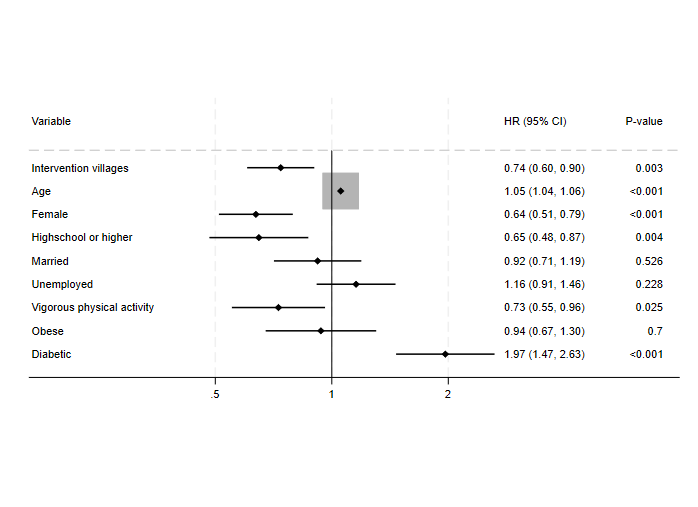

Supplement: S1 Fig — (TIF) [file pdig.0000476.s003.tif]

## A. Intervention villages

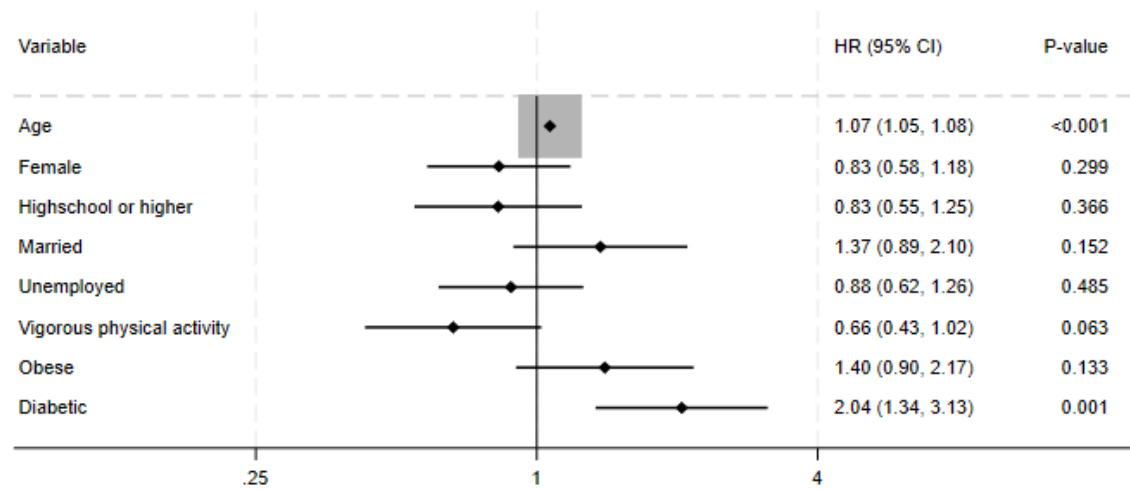

## B. Control villages

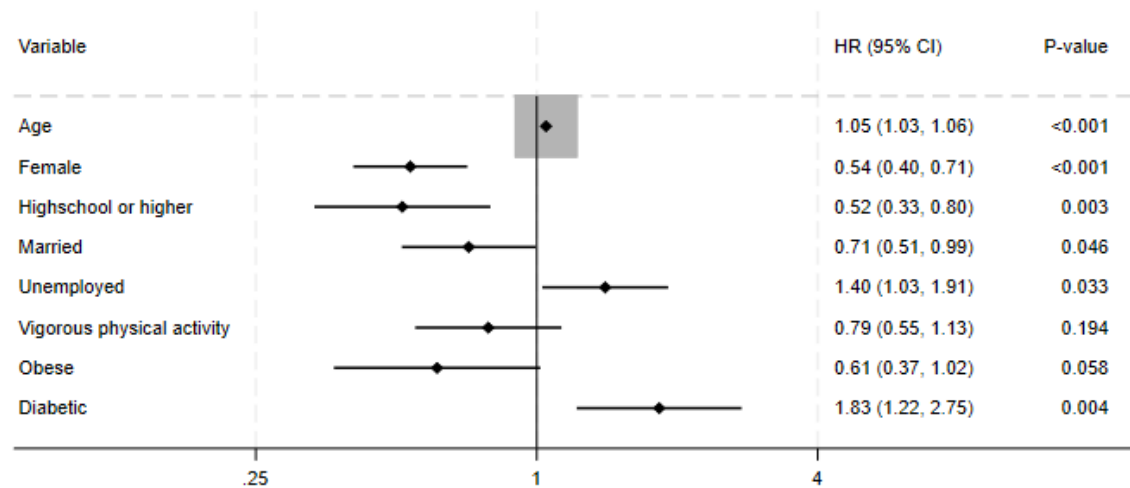

Supplement: S2 Fig — Cox proportional hazard models predicting the risk of CVD mortality in (A) intervention and (B) control villages. (PDF) [file pdig.0000476.s004.pdf]

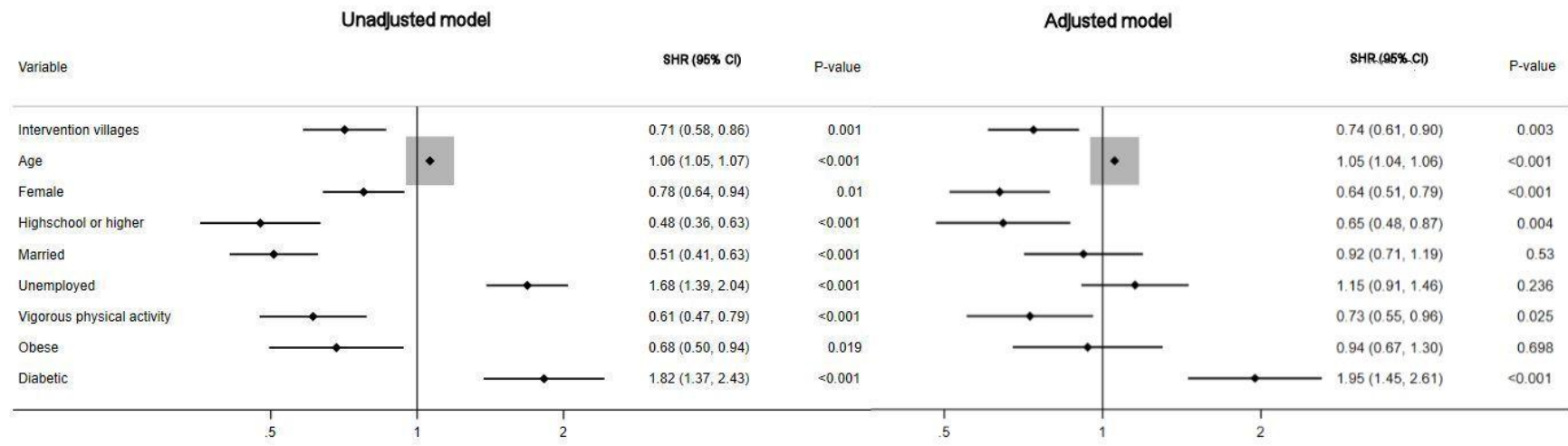

Supplement: S3 Fig — Subdistribution hazard ratios (95 CIs) for the association between intervention and cardiovascular mortality: (A) Unadjusted Model and (B) Adjusted Model. (PDF) [file pdig.0000476.s005.pdf]

## A. All villages

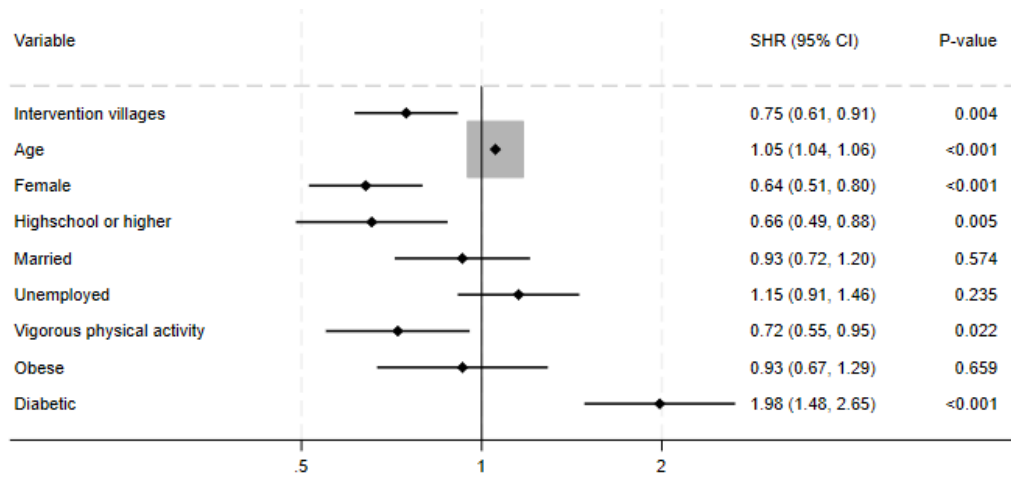

## B. Intervention villages

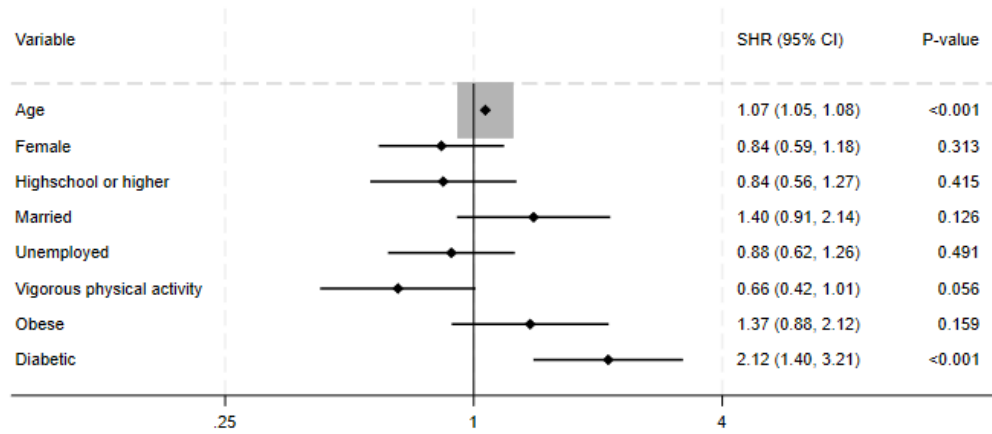

## C. Control villages

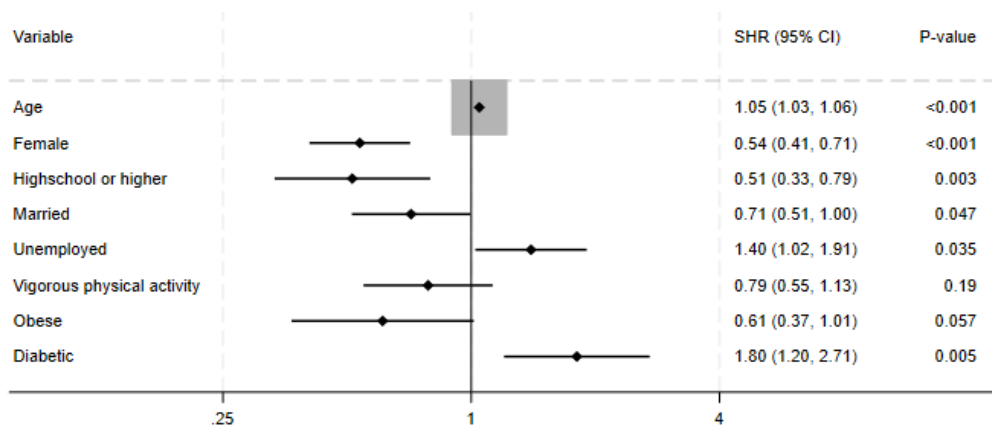

Supplement: S4 Fig — Fine and Gray subdistribution hazard of cardiovascular mortality in (A) all, (B) intervention and (C) control with the follow-up truncated on 2 March 2020. (PDF) [file pdig.0000476.s006.pdf]

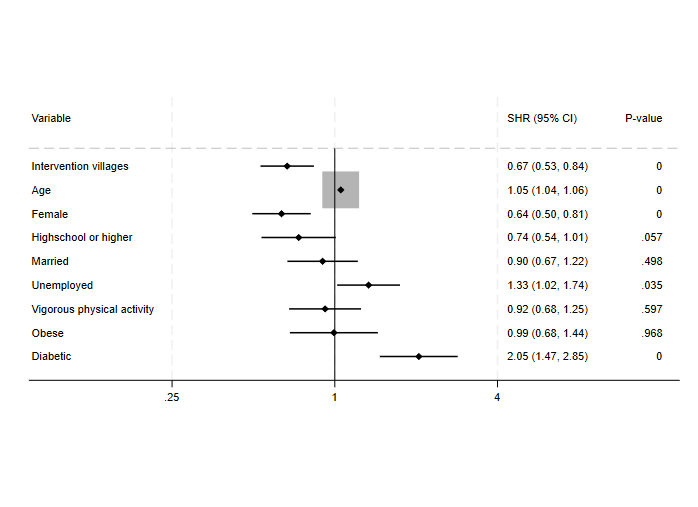

Supplement: S5 Fig — (TIF) [file pdig.0000476.s007.tif]
