# Supplementary material for: Implementation of mobile-health technology is associated with five-year survival among individuals in rural areas of Indonesia
Source: PLOS Digit Health. 2024 Apr 2;3(4):e0000476. doi: 10.1371/journal.pdig.0000476 (PMC10986960; doi:10.1371/journal.pdig.0000476)
Supplement: Attachment — Submitted filename: Response for Reviewers.docx [file pdig.0000476.s008.docx]

**Comments from Reviewer #1**

Clarify the data availability statement. I suspect you mean that confidential data is available through the George Institute, and that you will deposit the de-identified data in a repository. Right now, it’s not clear. You will need to provide the link to the publicly available repository.

**Authors’ response**

We thank you for this comment and suggestion. The data will be made available for academic and other researchers upon approval by the data custodian, i.e., the Malang District Health Agency (mortality) and The George Institute for Global Health (other data). We have included the following statement regarding the data availability (page 2, lines 23-28):

**“Data Availability:** The custodians of the data are the Malang District Health Agency (mortality) and The George Institute for Global Health (other data). Data and relevant meta-data will be made available to academic and other researchers after approval of a Data Access Request, subject to privacy and informed consent provisions, and in the case of mortality data, written approval from the data custodian. A request form can be obtained by email to the corresponding author or to DSC@georgeinstitute.org.”

**Comments from Reviewer #1**

Abstract:

The background should include a sentence from your introduction explaining the importance of the issue, e.g., “There is a pressing need to focus on implementing cost-effective health interventions and policies to reduce the cardiovascular disease burden in Indonesia.”

In the findings, the description of results is a little confusing, you probably meant to say something like this: “Participants residing in intervention villages were at 18% (95%CI = 4 to 30) lower risk of all-cause mortality.”

**Authors’ response**

Thank you for this comment. We have revised the Background section of the Abstract (page 3, lines 30-32) by adding the sentence, “There is an urgent need to focus on implementing cost-effective health interventions and policies to reduce the burden of cardiovascular disease in Indonesia.”

We have further revised the Findings section of the Abstract (page 3, lines 43-44) into “Participants residing in intervention villages were at 18% (95%CI = 4 to 30) lower risk of all-cause mortality.”

**Comments from Reviewer #1**

Introduction:

In the last sentence of the first paragraph, I think there should be the word “and” between smoking and physical activity.

**Authors’ response**

We have added the word “and” between smoking and physical activity on page 4, line 61.

**Comments from Reviewer #1**

I was confused about the distinction between SMARThealth Extend and SMARThealth. Please describe these in more detail.

**Authors’ response**

We have included the explanation on SMARThealth and SMARThealth Extend in the Introduction section on page 5 lines 77-84:

“SMARThealth is a multifaceted technology-based primary care intervention that enables kaders, nurses at the village health posts and physicians at the primary healthcare centres to collaborate in assessing an adult’s CVD risk using basic equipment and a clinical decision support app on a mobile device [7,8]. SMARThealth was implemented and evaluated in Indonesia through a study called SMARTHealth Extend. SMARThealth Extend was a quasi-experimental study that explored the potential of the intervention to identify and improve the care of people living with and at high risk of CVDs.”

**Comments from Reviewer #1**

Materials and methods:

Consider using more subheadings.

**Authors’ response**

Thank you for the input. We have included subheadings in the Materials and Methods section.

**Comments from Reviewer #1**

The sentence about the random blood sample (line 115) seems confusing. Consider taking out the words “were measured.”

Line 127, you say “Out of 22,103 individuals in the follow-up…” But I’m wondering if you mean the initial survey, since later you mentioned 17 individuals lost to follow-up.

On line 133, the word “one” is confusing. I think you mean to say that the determination of whether the death was recorded via verbal autopsy or not is unrelated to the risk factors or cause of death. This and the following sentences may be better off in the discussion section.

On line 135, do you mean “No physical autopsies were performed on the participants who died.”?

**Authors’ response**

Thank you for the detailed comments. We have revised the sentences in the Materials and Methods section:

- Line 129-130 (line 115 in prior draft) into “A random blood glucose sample was collected (with a record of the time since last ate) using the pin-prick method and portable glucometers (FreeStyle Optium Neo) [10].”.
- Line 139-140 (line 127 in prior draft) into: “Out of 22,103 individuals in the initial survey, 637 had died, and 17 were lost at follow-up (0.07%).”
- Lines 309-311 (line 133 in prior draft): We have revised the word “one” into “the” and moved the sentences into the Discussion section.
- Line 311-312 (line 135 in prior draft): We have revised the sentence into “No physical autopsies were performed on the participants who died”.

**Comments from Reviewer #1**

Results:

Figures

All the figures need legends. For example, Figure 1 needs a legend to distinguish between the two colors on the graphed lines. Please label the x axis. The “number at risk” is a bit confusing. You might want to explain in the text or remind people in a footnote to the figure that the reason it is 0 at month 60 is because that is the end of the follow-up timeframe. Also, in the text, you use the term “hazard ratio,” but the tables embedded in the figures use OR, or Odds Ratio. Explain in the text or in a footnote to the figure that HR is equivalent to OR, or change the headings on the tables. Please explain more about the horizontal bars in the figures. They appear to be a graphical description of the Hazard Ratio, but there’s no explanation in the figure itself or in a footnote below the figure. Figures 2 and 3 appear a bit blurry.

**Authors’ response**

Thank you for raising this point. We have revised all the figures by adding the legends and the footnote. We have also added the x-axis label for Figure 1. We have revised the “Odds Ratio” in Figure 2 and 3 into “Hazard Ratio” and increased the visibility of the Figures.

**Comments from Reviewer #1**

Discussion:

Line 257, please explain more about how the use of verbal autopsies strengthened the study.

**Authors’ response**

We have revised the sentence on page 15, lines 267-269 into “Our finding suggests that the methods used to determine the cause of death did not affect the relationship between the SMARThealth intervention and the risk of CVD mortality (Appendix 7).”

**Comments from Reviewer #1**

Lines 261 and 263, I was still a bit confused between SMARThealth Extend and SMARThealth intervention.

**Authors’ response**

Thank you for the comment. We have revised the sentence into “The SMARThealth intervention was related to higher awareness and use of BP-lowering medication [7].”

**Comments from Reviewer #1**

Line 269, I’m wondering if the word “association” would be better here than the word “effect.”

**Authors’ response**

We have revised the sentence on page 16, lines 279-280 into “Prior studies revealed the association between the female gender [15,16] and higher education attainment [17] with lower CVD mortality risks.”

**Comments from Reviewer #1**

On line 276, add the word “status” after “socio-economic.”

**Authors’ response**

We have added the word “status” after “socio-economic.”

**Comments from Reviewer #1**

On line 293, it might be clearer if you reworded to say “The first limitation is the unavailability of fasting blood glucose measurements from study participants.”

**Authors’ response**

We have revised the sentence to “The first limitation is the unavailability of fasting blood glucose measurements from study participants”.

**Comments from Reviewer #1**

On line 300, please re-word this sentence. It’s currently unclear.

**Authors’ response**

We have omitted the sentence.

**Comments from Reviewer #2**

Reviewer #2: I appreciate the opportunity to analyze this manuscript and I find the idea of using low-cost technology to improve access to healthcare very interesting, especially when they show such an impact as reducing mortality.

I suggest some changes and corrections below (following the order in which they appear in the text):

1. I believe there is a small typing error that hinders understanding in line 53

**Authors’ response**

Thank you for the comment. We have revised the sentence to “That prevalence varied among districts, with a higher prevalence of smoking and physical inactivity in rural areas.”.

**Comments from Reviewer #2**

2. in lines 61 and 62 two government programs are mentioned, I suggest putting the translation of the terms that, I believe, are in the Indonesian language

**Authors’ response**

We have included the translation of the term, i.e., Integrated Health Post for Non-Communicable Diseases.

**Comments from Reviewer #2**

3. in "Materials and methods" it was mentioned that sampling was not carried out since all eligible residents were invited. Even so, I suggest that a sample calculation be carried out to correctly determine the power of the study.

**Authors’ response**

We have included the sample size calculation on page 6, lines 104-108:

“Assuming a baseline rate of 10%, a cluster size of 144 individuals, and an intraclass correlation coefficient of 0.05, eight villages that were equally divided between the intervention and control groups were estimated to provide 80% power with a 2-sided α =.05 to detect an absolute difference of 18% in the proportion of high-risk individuals taking appropriate preventive medications.”

**Comments from Reviewer #2**

4. I also missed a baseline table comparing each variable in the 2 groups (intervention and control) with p value for the difference between the groups. This was almost done in table 1, but there is no comparison between the groups, only within each group between death and non-death. I understand that these differences were controlled in the following analyses, but I still think it is important to have a table that summarizes the differences between the control and intervention groups.

**Authors’ response**

We have added Appendix 2, comparing the p-value between intervention and control villages.

**Comments from Reviewer #3**

Reviewer #3: The paper aims evaluate whether a mobile technology–supported primary health care intervention, compared with usual care, would reduce the risk of all-cause mortality among people in rural Indonesia.

The overall quality of the study is very commendable.

The authors outlined the aims and methodology of the study.

The results were outlined and inferences from the data were clearly backed up with rigorous statistical analysis and robust discussions.

It also meets the requisite ethical standards.

The study is relevant and of significance in that it uses technology to improve access to the rural areas.

However, my main observation is on stretching (attributing) the outcome of the use of the SMARThealth App to reduction in “All-cause mortality’ instead of “CVD-related mortality” since SMARThealth (intervention) is CVD focused. This is more so that other cofounders that could have influenced mortalities from non-CVD causes (which is about two thirds of the cause of mortalities in the country) were not adequately controlled for.

**Authors’ response**

Thank you for the detailed comments. We have included the analysis predicting CVD-related mortality in Appendix 2-6.

**Comments from Reviewer #3**

I think it would be more appropriate to have the title of the study as Implementations of mobile-health technology is associated with lower CVD-related mortality after 5 years of follow-up in rural areas of Indonesia, and this aligns with the conclusion of the authors i.e. “In conclusion, our findings add to the literature by showing the potential technology-enabled primary care interventions in reducing the risk of CVD mortality in a rural Indonesian population (line 311-313)

It is however worth noting that changing the title does not necessarily change the interpretation that “A mobile technology–supported primary health care intervention had the potential to improve the five-year survival among people in the rural areas in an upper-middle income country.

The article is recommended for publication with proposed amendments.

**Authors’ response**

Thank you for the input. We revise the sentence in the Conclusion section into “In conclusion, our findings add to the literature by showing the potential for technology-enabled primary care interventions to reduce the risk of all-cause and CVD mortality in a rural Indonesian population.” We further revised the title to “Implementation of mobile-health technology is associated with five-year survival among individuals in rural areas of Indonesia”.
