# Supplementary material for: Implementation of mobile-health technology is associated with five-year survival among individuals in rural areas of Indonesia
Source: PLOS Digit Health. 2024 Apr 2;3(4):e0000476. doi: 10.1371/journal.pdig.0000476 (PMC10986960; doi:10.1371/journal.pdig.0000476)
Supplement: Attachment — Submitted filename: Response for Reviewers.docx [file pdig.0000476.s009.docx]

**Journal Requirements:**

Please review your reference list to ensure that it is complete and correct. If you have cited papers that have been retracted, please include the rationale for doing so in the manuscript text, or remove these references and replace them with relevant current references. Any changes to the reference list should be mentioned in the rebuttal letter that accompanies your revised manuscript. If you need to cite a retracted article, indicate the article's retracted status in the References list and also include a citation and full reference for the retraction notice.

**Authors’ response**

We have checked the references. There are no papers that have been retracted. However, there are two papers with corrections:

*Reference number 1:*

Roth GA, Mensah GA, Johnson CO, Addolorato G, Ammirati E, Baddour LM, et al. Global burden of cardiovascular diseases and risk factors, 1990–2019: update from the GBD 2019 study. J Am Coll Cardiol. 2020;76(25):2982-3021. doi: 10.1016/j.jacc.2020.11.010.

*The correction is:*

Correction, Journal of the American College of Cardiology, Volume 77, Issue 15,

2021, Pages 1958-1959, ISSN 0735-1097, https://doi.org/10.1016/j.jacc.2021.02.039.

(<https://www.sciencedirect.com/science/article/pii/S0735109721004629>).

*Reference number 18:*

Seshasai SRK, Kaptoge S, Thompson A, Angelantonio ED, Gao P, Sarwar N, Emerging Risk Factors Collaboration, et al. Diabetes mellitus, fasting glucose, and risk of cause-specific death. N Engl J Med. 2011;364(9):829-41. doi: 10.1056/NEJMoa1008862.

*The correction is:*

Correction, Diabetes Mellitus, Fasting Glucose, and Risk of Cause-Specific Death, N Engl J Med 2011; 364:1281. DOI: 10.1056/NEJMx110023

We follow the guideline in APA Style (<https://apastyle.apa.org/style-grammar-guidelines/research-publication/correction-notices#:~:text=Citing%20a%20corrected%20article,any%20errors%20from%20the%20original>), “Citing a corrected article

If you are citing an article that has been corrected, it is not necessary to note that the article has been corrected in the in-text citation or reference list entry. Simply write a standard reference list entry for the work, and ensure you do not reproduce any errors from the original. Readers will be informed of the correction when they retrieve the cited work.”

We ensure that we write the standard reference list entry for the work and that we do not reproduce any errors from the original.

**Additional Editor Comments (if provided):**

I appreciate the authors for their time and efforts to revise the manuscript. There are a few minor issues which need further attentions. I suggest you to follow the journal instructions for preparing the abstract and choosing appropriate headings and subheadings for the manuscript. Please add the “Research limitation” and “Conclusion” as headings, too.

**Authors’ response**

Thank you for the comments. We have followed the journal instructions and revised the headings and subheadings for the manuscript. We further added “Research limitation” and “Conclusion” as headings.

**Comments from Reviewer #1**

Check the requirements for the financial disclosure statement shown in the table on the first few pages prior to the manuscript. It looks like the Journal wants the funding statement to include author initials for who received the funding, the URL of the funder, and further details on the role of the funder in designing implementing and publishing the study results.

**Authors’ response**

We have revised the Financial Disclosure Statement:

SMARThealth Indonesia was funded by a grant from Give2Asia (https://give2asia.org/) on the recommendation of the Pfizer Foundation (AP, DP) and the Australian National Health and Medical Research (NHMRC) program (https://www.nhmrc.gov.au/) grant APP1052555 (AP, DP, GT, DO, AM, SJ). The funders had no role in study design, data collection and analysis, decision to publish, or preparation of the manuscript.
